# Personality and Survey Satisficing

**DOI:** 10.1093/poq/nfad036

**Published:** 2023-09-17

**Authors:** Patrick Sturgis, Ian Brunton-Smith

**Affiliations:** Professor, Department of Methodology, London School of Economics and Political Science, London, UK; Professor, Department of Sociology, University of Surrey, Guildford, UK

## Abstract

In this paper, we consider the role of personality as a component of motivation in promoting or inhibiting the tendency to exhibit the satisficing response styles of midpoint, straightlining, and Don’t Know responding. We assess whether respondents who are low on the Conscientiousness and Agreeableness dimensions of the Big Five Personality Inventory are more likely to exhibit these satisficing response styles. We find large effects of these personality dimensions on the propensity to satisfice in both face-to-face and self-administration modes and in probability and nonprobability samples. People who score high on Conscientiousness and Agreeableness were less likely to be in the top decile of straightlining and midpoint distributions. The findings for Don’t Know responding were weaker and only significant for Conscientiousness in the nonprobability sample. We also find large effects across all satisficing indicators for a direct measure of cognitive ability, where existing studies have mostly relied on proxy measures of ability such as educational attainment. Sensitivity analysis suggests the personality effects are likely to be causal in nature.

## Introduction

The theory of survey satisficing posits that a range of measurement errors in self-report questions arise from the inability or unwillingness of respondents to carry out the psychological operations necessary to provide high-quality answers (Krosnick and Alwin 1987; Krosnick 1991). Rather than adopting strategies that maximize the probability of a complete and accurate response, some respondents employ less costly approaches that yield “satisfactory” rather than “optimal” answers. The tendency to adopt a satisficing response strategy when answering a survey question is a function of three primary factors: the motivation and ability of the respondent and the difficulty of the task. Each of these factors is expected to exert an additive effect on the propensity to satisfice, though Krosnick has also suggested that multiplicative relationships are likely, such that, for example, the probability of a satisficing response is best characterized as an interaction between respondent ability and task difficulty (Krosnick, Narayan, and Smith 1996). In practice, however, the empirical literature has found weak support for this expectation, with only one in five studies that have considered multiplicative effects finding significant interactions (Roberts et al. 2019).

Given the longstanding recognition that personality seems likely to have an important role in shaping the propensity to satisfice (Krosnick 1991), it is surprising that no study has yet considered the dimensions of the Big Five Personality Inventory, which has become the dominant conceptual and empirical framework for personality measurement across the social sciences (Costa and McCrae 1992). The Big Five is derived from lexical analysis of comprehensive corpuses of natural language descriptors of enduring personality characteristics (McCrae and Costa 1994). These dimensions are Openness, Conscientiousness, Extraversion, Agreeableness, and Neuroticism. The dimensions of the Big Five are stable across the life course, and are strongly predictive of a very broad range of important life outcomes (Zell and Lesick 2022).

Our objective in this paper is to assess whether people who score low on the Conscientiousness and Agreeableness dimensions of the Big Five are more likely to exhibit satisficing response styles than otherwise similar individuals who score high on these dimensions. We expect people who score high on Conscientiousness to be less likely to provide the careless and ill-considered responses that typify satisficing. Respondents who score high on Agreeableness will, we expect, be more likely to adhere to the behavioral and conversational norms of the survey interview (Schober and Conrad 1997) by striving to provide accurate and complete responses to questions.

A secondary contribution of this paper is that we assess the role of cognitive ability in promoting satisficing using a comprehensive and direct measure of cognitive ability. In their systematic review of studies of satisficing, Roberts et al. (2019) found that 59 of 64 studies (92 percent) used a proxy measure of cognitive ability such as age, terminal age of education, and highest qualification. Of the five studies that these authors identified as using direct measures of cognitive ability, two were not based on general population samples and the remainder used measures of verbal ability only. In contrast, we use a general population sample and a measure of cognitive ability based on validated psychometric tests of memory, numerical, and verbal ability.

To foreshadow our key results, we find large, statistically significant effects of the Agreeableness and Conscientiousness personality dimensions on satisficing indicators in both face-to-face interview and online self-completion modes. Sensitivity analyses show that these effects are likely to be causal in nature. We also find that our measure of cognitive ability is a robust and powerful predictor of survey satisficing in both modes, suggesting that the mixed findings for cognitive ability in the existing literature are likely a result of reliance on proxy indicators. In the remaining sections of the paper, we first provide a review of the relevant literature, before describing the data and key measures used in our analysis. We then present our empirical results before concluding with a consideration of the limitations of our methodological approach, and the implications of our results for understanding of the psychology of survey response and for survey practice.

## Relevant Literature

The majority of studies that have assessed the motivational component of survey satisficing have used measures of self-reported interest in the topic of the survey (Roberts et al. 2019). For example, Tourangeau et al. (2009) found that respondents who expressed less interest in the survey topic provided more Don’t Know responses, a pattern also observed by Silber (2021), Robison (2015), and Enns and Richman (2013). Staszyńska (2011), likewise, found lower levels of acquiescence in political attitude questions among respondents who expressed more interest in politics, while Gummer and Roßmann (2015) found significantly longer response times on political attitude questions for respondents who expressed more interest in and engagement with politics.

Other scholars have produced similar findings using inferred rather than self-reported topic interest as the indicator of motivation. Chang and Krosnick (2009), for example, found that nonprobability online panelists were less likely to provide undifferentiated answers to adjacent questions compared to respondents drawn from probability samples, a difference that they concluded was due to the higher levels of political interest among the nonprobability panelists. Similarly, Callegaro et al. (2009) found longer response times for a survey completed by job applicants compared to job incumbents, which led them to conclude that the longer latencies for the job applicants reflected their greater motivation to complete the task accurately in order to present themselves in a favorable light to a prospective employer. Heerwegh and Loosveldt (2008) noted a similar difference between respondents who were required to log in to complete a web survey relative to a group who were logged in automatically, with the latter group providing significantly higher rates of item-missing data. The authors attributed this difference to the greater motivation of the respondents who had gone to the trouble of logging in, though whether this was a treatment effect of the log-in requirement or a compositional difference between samples due to nonresponse was unclear.

Researchers have also considered whether attitudes to the particular survey or to surveys in general might drive satisficing behavior. For example, Couper et al (2013) found that respondents who reported less interest in taking part in a survey were more likely to provide Don’t Know responses, though there was no difference in the degree of nondifferentiated responding between more and less interested respondents. Rogelberg et al. (2003) also found higher rates of item nonresponse among respondents who reported holding negative attitudes to surveys, while Tourangeau et al. (2009) found that those who expressed positive attitudes about the survey sponsor were less likely to provide nondifferentiated responses to adjacent attitude items.

Several studies have examined whether the position of an item in the questionnaire is related to the propensity to satisfice, under the assumption that respondent motivation declines as the number of questions they have answered increases. The results of these investigations have been mixed. Holbrook et al. (2007) found larger response order effects for items placed later in the questionnaire, and Krosnick et al. (2002) found that no-opinion responses increased in frequency as respondents approached the end of the questionnaire. In a later study, however, Holbrook et al. (2014) found no association between the position of an item in the questionnaire and the propensity to provide “heaped” responses to behavioral frequency questions, albeit that heaping does not appear to be a straightforward indicator of satisficing (see Turner et al. 2015).

Mode differences in the rate of satisficing have also been attributed to motivational factors, either because the presence of an interviewer is assumed to increase respondent motivation relative to self-completion (Chang and Krosnick 2009), or because respondents who were able to choose their preferred mode may be more motivated to optimize (Smyth, Olson, and Kasabian 2014). Other studies have used interviewer assessments of respondent cooperativeness during the interview as a proxy for motivation and found lower levels of satisficing among respondents rated as more cooperative (Kaminska, McCutcheon, and Billiet 2010; Kleiner, Lipps, and Ferrez 2015; Silber et al. 2021). For instance, Laurison (2015) found a 14 percent reduction in the rate of Don’t Know responding to political attitude items on the General Social Survey for each standard deviation increase in interviewer ratings of respondent cooperativeness. However, as Laurison notes, interviewer assessments of cooperativeness are likely endogenous to the rate of Don’t Know responding, which would serve to inflate effect sizes when using this type of measure.

A small but growing literature has found personality to be important in understanding survey errors, notably for nonresponse and attrition (Rogelberg et al. 2003; Lugtig 2014; Hübner et al. 2021). Survey methodologists have paid rather less attention to the role of personality in understanding measurement quality, despite a concern about the impact of individual differences on questionnaire responses stretching back as far as the 1940s (Cronbach 1946). There have been some mixed findings with regard to the Need to Evaluate and Need for Cognition traits (Jarvis and Petty 1996; Oppenheimer et al. 2009; Lenzner 2012; Leeper 2014). However, we are unaware of any existing study that has considered the Big Five personality dimensions as a cause of respondent propensity to satisfice. This is surprising because there are good *a priori* grounds for expecting that two dimensions of the Big Five should drive the propensity to satisfice. Conscientious individuals are hardworking, responsible and methodical, with an eye to detail and an achievement orientation, all factors that should lead them to devote more care and attention to the completion of questionnaires. Thus, our first hypothesis is:

**H1: People who score higher on Conscientiousness are less likely to satisfice.**

The Agreeableness dimension of the Big Five relates to an individual’s level of interpersonal warmth and ability to get along with others; the extent to which they are trustworthy, dependable, and oriented toward pro-social and communitarian action. Our second hypotheses is therefore:

**H2: People who score higher on Agreeableness are less likely to satisfice.**

Finally, because people with higher cognitive ability find it easier to process the information in survey questions and to carry out the cognitive operations required to provide a complete and accurate answer, our final hypothesis is:

**H3: People who score high on the measure of cognitive ability are less likely to satisfice.**

We see no clear theoretical reasons to expect that the remaining dimensions of the Big Five—Extraversion, Openness, and Neuroticism—should motivate respondents to provide complete and accurate answers to survey questions, so we do not include them in our analysis here. Considering subsets of dimensions of the Big Five Inventory is unproblematic because they are derived through factor analysis, such that each trait is orthogonal to the others (McCrae and Costa 1999). This is not to say that we expect these dimensions will be *unrelated* to satisficing but, rather, that the approach we are adopting here is to test *a priori* expectations rather than to conduct exploratory analyses.

## Data, Measures, and Analysis

The data for this study are drawn from two surveys: wave 3 of the UK Household Longitudinal Survey (UKHLS) and wave 1 of the British Election Study Online Panel (BESOP). The UKHLS is a multipurpose household panel survey covering topics of health, work, education, income, family, and social life. The survey has a multistage design with a stratified sample of postcode sectors, selected with probability proportional to their population size and with 18 households selected from each sector. All household members aged 16 and over are invited to provide an individual interview. Data collection for wave three of the UKHLS took place between January 2011 and July 2013, with interviews carried out face-to-face in respondents’ homes. Wave 3 of the survey is used because the questionnaire contains measures of the Big Five Personality Inventory and cognitive ability. A total of 49,692 individual interviews were conducted at wave 3, representing a net response rate of 32 percent from the wave 1 issued sample (Knies 2018). A total of 8,571 cases are dropped from the analysis because they did not complete the self-completion questionnaire, and we also exclude 3,830 proxy respondents which, combined with 474 cases with item nonresponse (no response recorded), yields an analysis sample of 36,817. The UKHLS questionnaire was interviewer-administered, but some questions were completed via Computer Assisted Self Interview (CASI). The results we present here combine the Computer Assisted Personal Interview (CAPI) and CASI items, but Supplementary Materialtables S1 and S2 present the same analyses conducted separately for the CAPI and CASI items. These show that, while there are some differences in the estimates, the broad pattern of findings is unchanged.

The BESOP is drawn from YouGov’s nonprobability online panel and has a sample size of 30,563 at wave 1 with fieldwork conducted between February 20 and March 9, 2014. This is reduced to 25,007 for our analysis sample because the Big Five personality measures were collected as part of an earlier YouGov survey, which 5,556 wave 1 BESOP respondents had not participated in. As a nonprobability sample, we are not able to report a response rate for this survey. The sample design uses quotas and calibration weighting to make the sample representative of the British general population on age, sex, region, social class, education, EU referendum vote, ethnic group, and political interest.

### Key Variables

The UKHLS and BESOP both include shortened versions of the Big Five Personality Inventory that were designed for survey administration and that have been used previously on both general population (Benet-Martínez and John 1998) and interviewer (Jäckle et al. 2013) samples. The UKHLS measures range from a minimum of 1 to a maximum of 7 (mean Conscientiousness = 5.5, Agreeableness = 5.6), while the BESOP measures range from a minimum of 0 to a maximum of 10 (mean Conscientiousness = 6.7, Agreeableness = 6.1).

The UKHLS contains measures of cognitive ability in the domains of memory, executive function, and numeric ability. There are four measures in total: (1) “delayed recall,” which assesses episodic memory by asking respondents to recall 10 words which they had previously read from a list, the score being the total number of words recalled correctly; (2) “serial subtraction,” which assesses working memory by providing the respondent with a number and asking them to subtract 7 from it, then subtract 7 again, and so on until 5 subtractions are completed, the final score being the total number of correct answers; (3) “verbal fluency,” which assesses executive function by asking respondents to name as many animals as possible in one minute, with the total score being equal to the number of animals mentioned; and (4) “numeric ability,” which assesses practical numeric knowledge by five questions of increasing mathematical difficulty. Further detail about the cognitive ability measures can be found in McFall (2013). For the sake of parsimony, we combine these four variables into a single measure of cognitive ability by taking the first factor in an exploratory factor analysis.^1^

The BESOP does not contain a direct measure of cognitive ability, so we use highest educational qualification as a proxy. We also include highest qualification as a covariate in the UKHLS models in order to assess whether a direct measure of cognitive ability and the standard proxy employed in the literature make independent contributions to satisficing. Political interest has been used as a measure of respondent motivation in a number of existing studies of survey satisficing (Roberts et al. 2019), so we also include this in both the UKHLS and BESOP models in addition to the personality variables. Controls are also included in both models for age and sex.

The dependent variables are three commonly used indicators of survey satisficing: midpoint responding, Don’t Know responding, and nondifferentiation (straightlining). For the UKHLS, these were derived from 46 items (33 CASI and 13 CAPI) and the BESOP measures were derived from 21 (self-administered) items. Question wordings for all items are provided in Appendix B. For the midpoint responding indicator, we take the count of midpoint responses for the items that included a midpoint. The same procedure is used for the Don’t Know indicator, with a count taken of all Don’t Know responses across all items. For the straightlining indicator, we first compute the mean root-of-pairs measure of nondifferentiation for contiguous items using the same response alternatives (Mulligan 1999) recommended by Kim et al. (2019). We then take the sum of the individual scale scores to produce an overall indicator of straightlining.

We take the top decile of each continuously derived measure as the indicator of satisficing and fit logistic regression models to predict the probability of being at the extreme end of the three measures. We do this because our interest is in respondents who give unusually high rates of these responses, as this is likely to reflect the use of these response styles as a way of reducing the cognitive burden of responding. For example, we would not expect the difference between one and two midpoint responses across a battery of 12 items to be very informative about whether the respondent is satisficing, as both would be considered within the “normal” range for such responses. On the other hand, we would be more confident that the difference between 10 and two midpoint responses is reflective of a genuine difference in satisficing behavior. Selecting the top decile is somewhat arbitrary, so we also fit models using the top 5 percent and 15 percent of each continuous measure, and these provide substantively identical results (see Supplementary Materialtables S3–S6). Treating the indicators as continuous in linear models also provides a broadly similar pattern of results (see Appendix Atables A1 and A2). Models fitted to the UKHLS account for the stratified sample design and the nonindependence of households within primary sampling units and of individuals within households. Estimates are weighted to account for differential selection probabilities, nonresponse at wave 1, and attrition between wave 1 and wave 3. The BESOP models apply calibration weights that adjust the sample to population totals for age, sex, region, and political interest. Models are estimated using Taylor-series linearization for variance estimation in Stata 15.1. Diagnostic tests do not indicate any problems arising from multicollinearity (VIF < 1.5 for all OLS models).

**Table 1.** Logistic regression models predicting response style indicators—UKHLS.

	Straightlining						Don’t knows						Midpoints					
	B	SE	Sig	B	SE	Sig	B	SE	Sig	B	SE	Sig	B	SE	Sig	B	SE	Sig
Agreeableness	−0.079	0.019	0.000	−0.095	0.019	0.000	−0.015	0.027	0.585	−0.028	0.027	0.300	−0.191	0.021	0.000	−0.193	0.021	0.000
Conscientiousness	−0.027	0.018	0.130	−0.036	0.018	0.045	−0.104	0.024	0.000	−0.040	0.025	0.106	−0.198	0.019	0.000	−0.166	0.020	0.000
Cognitive ability	−0.194	0.036	0.000	−0.085	0.042	0.042	−0.511	0.046	0.000	−0.397	0.058	0.000	−0.303	0.035	0.000	−0.304	0.044	0.000
Sex (female)				0.054	0.040	0.179				0.405	0.057	0.000				0.124	0.045	0.006
Interest in politics (ref = very)																		
Fairly interested				0.410	0.079	0.000				0.512	0.149	0.001				0.499	0.107	0.000
Not very interested				0.523	0.081	0.000				0.897	0.151	0.000				0.663	0.111	0.000
Not at all interested				0.452	0.085	0.000				1.590	0.148	0.000				0.796	0.111	0.000
Highest qualification (ref = degree)																		
Other degree				0.187	0.073	0.010				0.190	0.113	0.094				0.364	0.086	0.000
A-level				0.151	0.063	0.017				0.146	0.098	0.137				0.303	0.072	0.000
General Certificate of Secondary Education				0.319	0.061	0.000				0.365	0.097	0.000				0.475	0.074	0.000
Other qualification				0.263	0.080	0.001				0.540	0.116	0.000				0.520	0.094	0.000
No qualification				0.100	0.083	0.227				0.688	0.117	0.000				0.281	0.100	0.005
Age (years)				0.005	0.001	0.000				−0.024	0.002	0.000				−0.014	0.001	0.000
Constant	−1.630	0.121	0.000	−2.117	0.145	0.000	−2.118	0.158	0.000	−4.005	0.213	0.000	−0.377	0.127	0.003	−1.548	0.164	0.000
Unweighted N	36,817						36,817						36,817					

**Table 2.** Logistic regression models predicting response style indicators—BESOP.

	Straightlining						Don’t knows						Midpoints					
	B	SE	Sig	B	SE	Sig	B	SE	Sig	B	SE	Sig	B	SE	Sig	B	SE	Sig
Agreeableness	−0.043	0.016	0.007	−0.039	0.018	0.030	0.018	0.017	0.276	−0.001	0.020	0.963	−0.047	0.018	0.009	−0.042	0.019	0.025
Conscientiousness	−0.106	0.015	0.000	−0.065	0.017	0.000	−0.090	0.016	0.000	−0.050	0.018	0.006	−0.082	0.017	0.000	−0.073	0.017	0.000
Sex (female)				0.344	0.069	0.000				0.784	0.077	0.000				−0.143	0.069	0.039
Attention to politics				−0.240	0.013	0.000				−0.381	0.013	0.000				−0.121	0.012	0.000
Highest qualification (ref = postgraduate degree)																		
Undergraduate degree				0.157	0.142	0.269				0.299	0.153	0.051				−0.064	0.126	0.611
A-level				0.326	0.144	0.024				0.410	0.155	0.008				0.004	0.134	0.975
General Certificate of Secondary Education				0.729	0.144	0.000				0.553	0.156	0.000				0.203	0.135	0.131
Below General Certificate of Secondary Education				0.975	0.177	0.000				0.680	0.190	0.000				0.145	0.182	0.425
No qualification				0.992	0.169	0.000				0.602	0.183	0.001				−0.009	0.160	0.957
Age (years)				−0.036	0.002	0.000				−0.026	0.002	0.000				−0.003	0.002	0.133
Constant	−1.067	0.132	0.000	0.999	0.212	0.000	−1.803	0.129	0.000	0.435	0.223	0.051	−1.632	0.151	0.000	−0.775	0.214	0.000
Unweighted N	24,980						25,073						25,073					

To assess whether the coefficients for the personality variables can be interpreted as causal effects, we use a form of sensitivity analysis that determines whether the presence of an unobserved confounder could render the coefficients of interest statistically nonsignificant (Cinelli and Hazlett 2020). Based on the omitted variable bias framework, the logic of this approach is to first identify plausible bounds for the magnitude of an unobserved confounder by benchmarking against theoretically selected predictor variables that are included in the model (see Ansell et al. 2022; Fell et al. 2022 for examples of recent applications of this method). The test is then based on an assessment of how the coefficients of interest, in our case the personality measures, would change if a predictor variable that explains as much of the residual variation in the outcome as this benchmark (or multiples of it) were added to the model. We examine scenarios where an unobserved causal effect is at least as large as the magnitude of the effects observed for cognitive ability (for the UKHLS data) and for political attentiveness (for the BESOP data) for 1, 2, and 3 times the magnitude of these effects.

## Results

Table 1 presents estimates from the logistic regression models for the UKHLS. For each satisficing indicator, there are two models; the first includes the two personality and cognitive ability measures, and the second adds the indicators of motivation and the covariates. The coefficients in table 1 are log odds ratios (logits). For all three satisficing indicators, the coefficient for cognitive ability is significant and in the expected direction; higher cognitive ability is associated with a lower propensity to satisfice, though the effect size is larger for Don’t Knows and midpoints than for straightlining. The coefficients for educational qualifications are also in the expected direction, with graduates least likely to satisfice on all three indicators.

The fact that the cognitive ability measure is significant when controlling for highest qualification suggests that education is, as we might expect, a rather inexact proxy for cognitive ability. The estimates for political interest, too, are significant and in line with theoretical expectation, with those who express less interest in politics (and who are therefore less motivated to answer the questions) having a higher probability of satisficing on all three indicators. Women are more likely to provide high rates of Don’t Know and midpoint responses than men, but there is no sex difference in the propensity to straightline. The older people are, the more likely they are to straightline, though the opposite is true for Don’t Know and midpoint responding.

**Figure 1.**
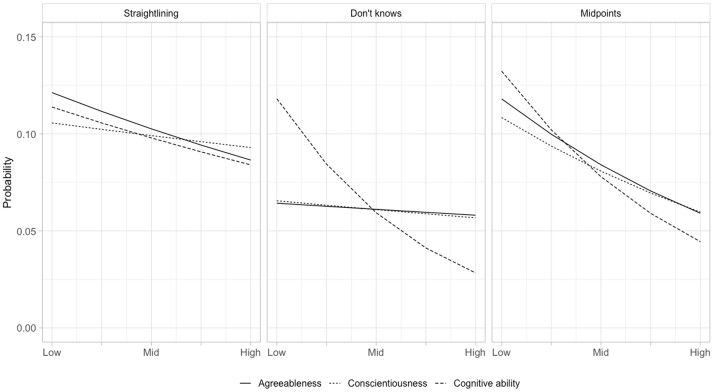
Marginal effects of Agreeableness and Conscientiousness on probability of satisficing behaviors, UKHLS. Data: UKHLS, Wave 3, n = 36,817.

For both personality measures, the results are significant and in the expected direction for straightlining and midpoint responding in the models including covariates, but neither Conscientiousness nor Agreeableness is significantly related to Don’t Know responding in the model with controls. These relationships can be seen more clearly in figure 1, which plots the marginal effect of unit changes in each personality dimension and cognitive ability on the probability of satisficing for each indicator, holding the covariates at their mean/reference values.^2^ The strongest association with personality is for midpoints, with the probability of being in the top decile of midpoint responding nearly doubling from 0.06 to 0.12 moving from the 5th to the 95th percentile on both Conscientiousness and Agreeableness.

For straightlining, the relationship with Conscientiousness is weak, with only a small difference between those at the bottom (0.09) and those at the top (0.10) of the scale. For Agreeableness, the relationship is stronger, with the probability of being in the top decile of straightlining increasing from 0.09 to 0.12 between the lowest and highest points on the Agreeableness measure. That these relationships are stronger for Agreeableness than for Conscientiousness suggests that Agreeableness might play a more important role in the context of a face-to-face interview than in self-completion, perhaps not a surprising pattern given the conversational nature of an in-person interview.

Table 2 presents the corresponding results for the BESOP models, which show essentially the same pattern of findings. Both Conscientiousness and Agreeableness are associated with straightlining and midpoint responding in the expected directions. As with the UKHLS, Agreeableness is not associated with Don’t Know responding, but Conscientiousness is now weakly correlated with this indicator in both the unconditional model and the model with covariates. The covariate patterns are also similar, though not identical to those found for the UKHLS, albeit there is no corresponding direct measure of cognitive ability in the BESOP models, so they are not directly comparable.

Political interest is again consistently associated with all three satisficing indicators, with less politically engaged individuals more likely to satisfice. Higher qualification attainment is again predictive of both straightlining and Don’t Know responding, although not for the midpoint satisficing indicator. Age and sex follow the same pattern as for the UKHLS on Don’t Knows, with women and younger people more likely to be in the top decile for this indicator. However, for straightlining, age has the opposite sign than was found for the UKHLS, older people being less likely to exhibit high levels of nondifferentiation in the nonprobability survey. The age and sex pattern is also discordant between surveys for midpoint responding, with women in the BESOP less likely to satisfice on this indicator, while the coefficient for age is not significantly different from zero.

**Figure 2.**
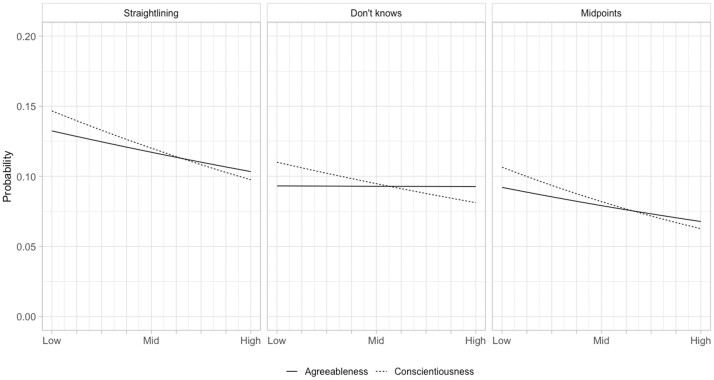
Marginal effects of Agreeableness and Conscientiousness on probability of satisficing behaviors, BESOP. Data: BESOP, n = 25,007.

Figure 2 shows the marginal effect plots from the models in table 2, which reinforces visually the similarity of the findings between the two surveys. As was the case for the UKHLS, the weakest effects are for Don’t Know responding, although there is a small but significant effect in the expected direction for Conscientiousness in the BESOP. While varying somewhat in magnitude, all other effects are consistent between surveys, with people who score high on both Conscientiousness and Agreeableness more likely to be in the top decile for straightlining and midpoint responding. The strength of the relationship between Agreeableness and straightlining/midpoint responding is somewhat stronger in the UKHLS, which suggests that this dimension may be a more important driver of satisficing in an interviewer-administered than a self-completion survey. Overall, however, what is most striking about the plots in figures 1 and 2 is the similarities rather than the differences across surveys.

The results of the causal sensitivity analyses are shown in table 3. Models are only estimated for personality effects that are statistically significant in tables 1 and 2. The results strongly suggest that these effects are causal in nature, with the significant effects of Agreeableness and Conscientiousness unchanged, even if there is an unobserved confounder with effects on the satisficing indicators that are at least as strong as cognitive ability and political attentiveness. Indeed, these sensitivity analyses suggest that most of these personality effects would still be large and statistically significant in the presence of unobserved confounders that are three times as large as the effects of cognitive ability and political attentiveness (these additional estimates are included in Supplementary Materialtables S7 and S8).

**Table 3.** Causal sensitivity analysis results.^a^

	Agreeableness			Conscientiousness		
	B	SE	Sig	B	SE	Sig
*UKHLS*						
Straightlining						
Original estimate	−0.009	0.002	0.000	−0.0033	0.002	0.026
1x cognitive ability	−0.008	0.002	0.000	−0.0031	0.002	0.037
Midpoints						
Original estimate	−0.015	0.001	0.000	−0.0131	0.001	0.000
1x cognitive ability	−0.015	0.001	0.000	−0.0125	0.001	0.000
Unweighted N	36,817			36,817		
*BESOP*						
Straightlining						
Original estimate	−0.004	0.001	0.001	−0.0058	0.001	0.000
1x political attentiveness	−0.004	0.001	0.001	−0.0050	0.001	0.000
Don’t knows						
Original estimate				−0.0031	0.001	0.001
1x political attentiveness				−0.0020	0.001	0.033
Midpoints						
Original estimate	−0.003	0.001	0.004	−0.0053	0.001	0.000
1x political attentiveness	−0.003	0.001	0.005	−0.0050	0.001	0.000
Unweighted N	25,073			25,073		

^a^ Models are estimated using the R sensemakr package using a linear probability model.

## Discussion

Our objective in this paper has been to assess the utility of the Conscientiousness and Agreeableness dimensions of the Big Five Personality Inventory as measures of respondent motivation in survey completion. Additionally, we have tested the effect of a direct measure of cognitive ability on the propensity to satisfice. Our results show large and statistically significant effects in the expected directions for both personality dimensions and for cognitive ability across face-to-face and online self-completion modes and using probability and nonprobability samples for midpoint and straightlining indicators. These findings provide strong support for our first and second hypotheses and suggest that the Conscientiousness and Agreeableness dimensions of the Big Five are valid and useful measures of respondent motivation across survey contexts. Our results are consistent with the theoretical expectations derived from personality theory; Conscientious individuals tend to be responsible and methodical, while those who score high on Agreeableness tend to be trustworthy, dependable, and oriented toward pro-social action. These orientations, we conclude, lead respondents possessing these personality types to devote more care and attention to the completion of questionnaires.

There is some suggestion in the pattern of results that the effects of Agreeableness on satisficing are stronger in the face-to-face than the self-completion interview mode, although this requires further research and a greater coverage of items across the respective modes. Hypothesis 3, that satisficing responses are less frequent for respondents with higher cognitive ability, is also supported on all indicators. The magnitude and significance of these results also suggest that Conscientiousness and Agreeableness are stronger predictors of satisficing than Need for Cognition and Need to Evaluate, which have been the most frequently studied measure of personality in the existing literature, although additional research is also needed to establish this satisfactorily.

The results for Don’t Know responding were more mixed, with a significant coefficient of moderate size for Conscientiousness but no significant effect for Agreeableness in the BESOP and with both personality variables nonsignificant in the UKHLS. This pattern suggests that Don’t Know responding may not be a good indicator of satisficing across survey contexts, with the tendency to provide Don’t Know responses more reflective of genuine respondent uncertainty, particularly in face-to-face interviews (Sturgis et al. 2008; Turner et al. 2015).

In assessing the generality of our findings, it is worth noting that we did not consider acquiescent responding as a satisficing indicator, because we were not able to derive a satisfactory measure of it from the items available in the UKHLS and BESOP waves used in our analysis. This is a potentially important omission because there are good reasons to think that Agreeableness may have an opposite signed relationship for this behavior, which is to say that people who score higher on Agreeableness will be *more* likely to exhibit an acquiescent response style. That being said, however, it may be questioned whether such a positive relationship between Agreeableness and acquiescence would be due to satisficing. For satisficing to be the cause of such an association would require that more Agreeable respondents disproportionately choose “agree” and “yes” options *in order to save cognitive costs*. However, if such a positive association exists, it would seem more likely to arise as a result of Agreeable people preferring to agree with others, over disagreeing. In any event, the nature of the relationship between Agreeableness and the tendency to exhibit an acquiescent response style is a subject worthy of further attention.

Future research might also consider how the Extraversion, Openness, and Neuroticism dimensions of the Big Five are related to satisficing behavior. The approach we have taken here has been deductive, testing *a priori* hypotheses derived from theoretical expectations about how specific dimensions of personality are related to indicators of satisficing. No clear theoretical expectations are evident for Extraversion, Openness, and Neuroticism, so we did not test hypotheses for them. This does not, however, imply an expectation that these relationships are zero, and exploratory work considering these (and other) personality traits could usefully advance understanding in this area.

While the personality effects we have observed here are large, there are several reasons to believe that we may be underestimating their true magnitude. First, a nonprobability online panel and the third wave of a face-to-face probability panel survey are likely to underrepresent people who are weakly motivated to complete surveys which will, in turn, understate the effect of measures of motivation on satisficing. Second, our use of short scales to measure the Big Five personality dimensions means that random measurement error in these variables will bias the coefficient estimates toward zero compared to longer multi-item batteries (Bakker and Lelkes 2018). Third, some of the effects of the personality variables on the propensity to satisfice may be indirect, via their direct effects on mediating variables in the models, such as political interest and education. In any event, irrespective of their true magnitude, our analysis suggests the effects we have observed are likely to be causal in nature.

To the best of our knowledge, this study is also the first to use a direct measure of cognitive ability as a predictor of satisficing on a general population sample. Using a multi-item measure covering memory, numerical, and verbal ability, we found statistically significant and substantively large effects when predicting straightlining, midpoint, and Don’t Know responding. Our models controlled for highest educational qualification, which also had large and statistically significant effects, demonstrating that educational attainment and cognitive ability are related but distinct concepts. These findings, then, suggest that the failure to detect significant effects of cognitive ability on satisficing in close to half of existing studies likely results from a reliance on weak proxy measures of cognitive ability. On the other hand, it should also be acknowledged that the measure of cognitive ability we used has a high level of task difficulty for some respondents, as it requires a series of cognitively demanding operations using numbers and words. It is therefore likely that respondents who are prone to satisficing will score lower on these items, inflating somewhat the association between the cognitive ability measure and the indicators of satisficing. This may, then, offset, at least in part, the errors that serve to weaken the cognitive ability/satisficing correlation noted above. Neither have we considered which dimensions of cognitive ability are most important in underpinning different satisficing response styles, an important question that we leave to future research stimulated by these findings.

There is, of course, little that survey researchers can do to alter the personalities of respondents in an effort to improve response quality. Nonetheless, a potential lesson for improving survey practice is that administering personality measures in the first wave of a panel study might open up the possibility of implementing tailored interventions to respondents who are low on the relevant personality dimensions, with the goal of reducing their propensity to use satisficing response styles in later waves. Our findings also have important implications for satisficing theory and its role in shaping measurement quality in surveys. They suggest that greater care is needed on the part of researchers in selecting measures of both satisficing and the respondent characteristics that give rise to these response effects, in order to avoid false negative findings. Using theoretically appropriate, direct measures of respondent ability and personality produced substantively strong, statistically significant effects that are consistent with theoretical expectation.

## Supplementary Material

nfad036_Supplementary_Data

## Data Availability

Replication data and documentation are available at: https://osf.io/z9tng/.

## Footnotes

1 The four variables are moderately positively correlated (range = 0.19–0.37), and the first factor has an eigenvalue of 1.5 and the second factor an eigenvalue of 0.07. The factor loadings are 0.5 (delayed recall), 0.46 (serial subtraction), 0.55 (verbal fluency), and 0.61 (numeric ability).

2 Marginal effects are calculated from the models including covariates.

## Appendixes

### Appendix A. Linear Regression Models

**Table A1.** Linear regression models predicting response style indicators—UKHLS.

	Straightlining						Don’t knows						Midpoints					
	B	SE	Sig	B	SE	Sig	B	SE	Sig	B	SE	Sig	B	SE	Sig	B	SE	Sig
Agreeableness	−0.010	0.002	0.000	−0.013	0.002	0.000	−0.002	0.004	0.542	−0.004	0.004	0.299	−0.393	0.022	0.000	−0.402	0.022	0.000
Conscientiousness	0.005	0.002	0.008	0.002	0.002	0.419	−0.020	0.004	0.000	−0.012	0.004	0.003	−0.415	0.021	0.000	−0.368	0.021	0.000
Cognitive ability	−0.001	0.004	0.875	0.029	0.004	0.000	−0.069	0.007	0.000	−0.057	0.009	0.000	−0.055	0.041	0.181	−0.185	0.046	0.000
Sex (female)				0.000	0.004	0.969				0.052	0.007	0.000				0.244	0.043	0.000
Interest in politics (ref = very)																		
Fairly interested				0.083	0.007	0.000				−0.002	0.006	0.807				0.696	0.071	0.000
Not very interested				0.098	0.008	0.000				0.026	0.008	0.002				0.952	0.076	0.000
Not at all interested				0.082	0.009	0.000				0.210	0.014	0.000				0.987	0.084	0.000
Highest qualification (ref = degree)																		
Other degree				0.033	0.008	0.000				0.021	0.012	0.071				0.362	0.071	0.000
A-level				0.027	0.007	0.000				0.001	0.010	0.927				0.388	0.063	0.000
GCSE				0.058	0.007	0.000				0.046	0.012	0.000				0.546	0.068	0.000
Other qualification				0.034	0.009	0.000				0.074	0.016	0.000				0.573	0.090	0.000
No qualification				0.011	0.010	0.263				0.076	0.016	0.000				0.048	0.089	0.589
Age (years)				0.002	0.000	0.000				−0.004	0.000	0.000				−0.026	0.001	0.000
Constant	3.044	0.014	0.000	2.975	0.016	0.000	0.258	0.027	0.000	0.104	0.026	0.000	11.239	0.155	0.000	9.823	0.164	0.000
Unweighted N	36,817						36,817						36,817					

**Table A2.** Linear regression models predicting response style indicators—BESOP.

	Straightlining						Don’t knows						Midpoints					
	B	SE	Sig	B	SE	Sig	B	SE	Sig	B	SE	Sig	B	SE	Sig	B	SE	Sig
Agreeableness	−0.003	0.003	0.247	−0.005	0.003	0.066	0.037	0.019	0.048	−0.014	0.018	0.442	−0.050	0.016	0.002	−0.041	0.017	0.013
Conscientiousness	−0.022	0.003	0.000	−0.010	0.003	0.000	−0.077	0.019	0.000	−0.004	0.017	0.797	−0.072	0.016	0.000	−0.069	0.016	0.000
Sex (female)				0.088	0.010	0.000				0.982	0.065	0.000				−0.222	0.060	0.000
Attention to politics				−0.074	0.002	0.000				−0.589	0.017	0.000				−0.109	0.014	0.000
Highest qualification (ref = postgraduate degree)																		
Undergraduate degree				0.046	0.016	0.005				0.273	0.107	0.011				−0.077	0.100	0.439
A-level				0.071	0.018	0.000				0.478	0.116	0.000				−0.233	0.108	0.031
GCSE				0.152	0.018	0.000				0.579	0.113	0.000				−0.054	0.112	0.628
Below GCSE				0.196	0.026	0.000				0.571	0.162	0.000				−0.053	0.156	0.734
No qualification				0.173	0.021	0.000				0.635	0.138	0.000				−0.463	0.127	0.000
Age (years)				−0.007	0.000	0.000				−0.026	0.002	0.000				0.001	0.002	0.444
Constant	1.535	0.024	0.000	2.123	0.031	0.000	2.570	0.151	0.000	6.576	0.222	0.000	5.610	0.139	0.000	6.442	0.196	0.000
Unweighted N	24,980						25,073						25,073					

## Appendix B. Question Wordings

UKHLS CASI questions

All UKHLS items listed below were used to derive the counts of midpoint and Don’t Know responses and to derive the straightlining indicators. The items in the GHQ scale do not include a midpoint, so these items do not contribute to the midpoint satisficing indicator.

SF12 items

*During the past 4 weeks, how much of the time have you had any of the following problems with your work or other regular daily activities as a result of your physical health?*

1. Accomplished less than you would like.

All of the time, most of the time, some of the time, a little of the time, none of the time

2. Were limited in the kind of work or other activities.

All of the time, most of the time, some of the time, a little of the time, none of the time

During the past 4 weeks, how much of the time have you had any of the following problems with your work or other regular daily activities as a result of any emotional problems?

3. Accomplished less than you would like.

All of the time, most of the time, some of the time, a little of the time, none of the time

4. Did work or other activities less carefully than usual.

All of the time, most of the time, some of the time, a little of the time, none of the time

5. During the past 4 weeks, how much did pain interfere with your normal work (including both work outside the home and housework)?

Not at all, a little bit, moderately, quite a bit, extremely

*How much of the time during the past 4 weeks …*

6. Have you felt calm and peaceful?

All of the time, most of the time, some of the time, a little of the time, none of the time

7. Did you have a lot of energy?

All of the time, most of the time, some of the time, a little of the time, none of the time

8. Have you felt downhearted and depressed?

All of the time, most of the time, some of the time, a little of the time, none of the time

9. During the past 4 weeks, how much of the time has your physical health or emotional problems interfered with your social activities (like visiting friends, relatives, etc.)?

All of the time, most of the time, some of the time, a little of the time, none of the time

GHQ Scale

1. Have you recently been able to concentrate on whatever you’re doing?

Better than usual, same as usual, less than usual, much less than usual

2. Have you recently lost much sleep over worry?

Not at all, no more than usual, rather more than usual, much more than usual

3. Have you recently felt that you were playing a useful part in things?

More than usual, same as usual, less so than usual, much less than usual

4. Have you recently felt capable of making decisions about things?

More so than usual, same as usual, less so than usual, much less capable

5. Have you recently felt constantly under strain?

Not at all, no more than usual, rather more than usual, much more than usual

6. Have you recently felt you couldn’t overcome your difficulties?

Not at all, no more than usual, rather more than usual, much more than usual

7. Have you recently been able to enjoy your normal day-to-day activities?

More than usual, same as usual, less so than usual, much less than usual

8. Have you recently been able to face up to problems?

More so than usual, same as usual, less able than usual, much less able

9. Have you recently been feeling unhappy or depressed?

Not at all, no more than usual, rather more than usual, much more than usual

10. Have you recently been losing confidence in yourself?

Not at all, no more than usual, rather more than usual, much more than usual

11. Have you recently been thinking of yourself as a worthless person?

Not at all, no more than usual, rather more than usual, much more than usual

12. Have you recently been feeling reasonably happy, all things considered?

More than usual, same as usual, less so than usual, much less than usual

Neighborhood belonging items

1. I feel like I belong in this neighborhood.

Strongly agree, agree, neither agree/disagree, disagree, strongly disagree

2. The friendships and associations I have with other people in my neighborhood mean a lot to me.

Strongly agree, agree, neither agree/disagree, disagree, strongly disagree

3. If I needed advice about something, I could go to someone in my neighborhood.

Strongly agree, agree, neither agree/disagree, disagree, strongly disagree

4. I borrow things and exchange favors with my neighbors.

Strongly agree, agree, neither agree/disagree, disagree, strongly disagree

5. I would be willing to work together with others on something to improve my neighborhood.

Strongly agree, agree, neither agree/disagree, disagree, strongly disagree

6. I plan to remain a member of this neighborhood for a number of years.

Strongly agree, agree, neither agree/disagree, disagree, strongly disagree

7. I think of myself as similar to people that live in this neighborhood.

Strongly agree, agree, neither agree/disagree, disagree, strongly disagree

8. I regularly shop and talk with people in my neighborhood.

Strongly agree, agree, neither agree/disagree, disagree, strongly disagree

Life satisfaction items

*Please choose the number which you feel best describes how satisfied or dissatisfied you are with the following aspects of your current situation.*

1. Your life overall

Completely dissatisfied, mostly dissatisfied, somewhat dissatisfied, neither sat nor dissat, mostly satisfied, completely satisfied

2. Your health

Completely dissatisfied, mostly dissatisfied, somewhat dissatisfied, neither sat nor dissat, mostly satisfied, completely satisfied

3. The income of your household

Completely dissatisfied, mostly dissatisfied, somewhat dissatisfied, neither sat nor dissat, mostly satisfied, completely satisfied

4. The amount of leisure time you have

Completely dissatisfied, mostly dissatisfied, somewhat dissatisfied, neither sat nor dissat, mostly satisfied, completely satisfied

UKHLS CAPI questions

Social capital items

1. People around here are willing to help their neighbors.

Strongly agree, agree, neither agree/disagree, disagree, strongly disagree

2. This is a close-knit neighborhood.

Strongly agree, agree, neither agree/disagree, disagree, strongly disagree

3. People in this neighborhood can be trusted.

Strongly agree, agree, neither agree/disagree, disagree, strongly disagree

4. People in this neighborhood generally don’t get along with each other.

Strongly agree, agree, neither agree/disagree, disagree, strongly disagree

5. I would be seriously neglecting my duty as a citizen if I didn’t vote.

Strongly agree, agree, neither agree/disagree, disagree, strongly disagree

6. Voting is a good way to get benefits for groups that people care about, like pensioners or the disabled.

Strongly agree, agree, neither agree/disagree, disagree, strongly disagree

7. I feel a sense of satisfaction when I vote.

Strongly agree, agree, neither agree/disagree, disagree, strongly disagree

8. It takes too much time to be active in politics and public affairs.

Strongly agree, agree, neither agree/disagree, disagree, strongly disagree

9. Most people around here usually vote in general elections.

Strongly agree, agree, neither agree/disagree, disagree, strongly disagree

10. I consider myself to be well qualified to participate in politics.

Strongly agree, agree, neither agree/disagree, disagree, strongly disagree

11. I think I am better informed about politics than most people.

Strongly agree, agree, neither agree/disagree, disagree, strongly disagree

12. Public officials don’t care much about what people like me think.

Strongly agree, agree, neither agree/disagree, disagree, strongly disagree

13. People like me don’t have any say in what government does.

Strongly agree, agree, neither agree/disagree, disagree, strongly disagree

UKHLS Big Five items (the Big Five items were not used to derive the satisficing indicators)

*The following questions are about how you see yourself as a person. Please choose the number which best describes how you see yourself, using a scale from 1 to 7 where 1 means “does not apply to me at all” and 7 means “applies to me perfectly.”*

Agreeableness items

I see myself as someone who is sometimes rude to others.

I see myself as someone who has a forgiving nature.

I see myself as someone who is considerate and kind to almost everyone.

Conscientiousness items

I see myself as someone who does a thorough job.

I see myself as someone who tends to be lazy.

I see myself as someone who does things efficiently.

Extraversion items

I see myself as someone who is talkative.

I see myself as someone who is outgoing, sociable.

I see myself as someone who is reserved.

Neuroticism items

I see myself as someone who worries a lot.

I see myself as someone who gets nervous easily.

I see myself as someone who is relaxed, handles stress well.

Openness items

I see myself as someone who is original, comes up with new ideas.

I see myself as someone who values artistic, aesthetic experiences.

I see myself as someone who has an active imagination.

BESOP Questions

All BESOP items listed below were used to derive the counts of midpoint and Don’t Know responses and to derive the straightlining indicators.

Government policy items

*Some people feel that government should make much greater efforts to make people’s incomes more equal. Other people feel that government should be much less concerned about how equal people’s incomes are. Where would you place yourself and the political parties on this scale?*

1. Yourself

Government should try to make incomes more equal 0, 1, 2 … 10 Government should be less concerned about equal incomes

2. The Conservatives

Government should try to make incomes more equal 0, 1, 2 … 10 Government should be less concerned about equal incomes

3. Labour

Government should try to make incomes more equal 0, 1, 2 … 10 Government should be less concerned about equal incomes

4. Liberal Democrats

Government should try to make incomes more equal 0, 1, 2 … 10 Government should be less concerned about equal incomes

5. UKIP

Government should try to make incomes more equal 0, 1, 2 … 10 Government should be less concerned about equal incomes

*If there were a *Labour* government today, do you think that each of the following would be getting better, getting worse, or staying about the same?*

6. The economy

Getting a lot worse, getting a little worse, staying about the same, getting a little better, getting a lot better

7. The NHS

Getting a lot worse, getting a little worse, staying about the same, getting a little better, getting a lot better

8. Schools

Getting a lot worse, getting a little worse, staying about the same, getting a little better, getting a lot better

*If there were a *Labour* government today, do you think that each of the following would be getting higher, getting lower, or staying about the same?*

9. The cost of living

Getting a lot lower, getting a little lower, staying about the same, getting a little higher, getting a lot higher

10. The level of immigration

Getting a lot lower, getting a little lower, staying about the same, getting a little higher, getting a lot higher

11. The level of crime

Getting a lot lower, getting a little lower, staying about the same, getting a little higher, getting a lot higher

Coalition government items

*Thinking about coalition government, how much do you agree or disagree with the following statements?*

1. It is more difficult to know who to blame when parties govern in coalition.

Strongly agree, agree, neither agree/disagree, disagree, strongly disagree

2. Coalition governments are more effective than single-party governments.

Strongly agree, agree, neither agree/disagree, disagree, strongly disagree

3. Coalition governments are more in tune with the public than governments formed of one party.

Strongly agree, agree, neither agree/disagree, disagree, strongly disagree

4. Parties cannot deliver on their promises in coalition.

Strongly agree, agree, neither agree/disagree, disagree, strongly disagree

Political efficacy items

*How much do you agree or disagree with the following statements?*

1. It is every citizen’s duty to vote in an election.

Strongly agree, agree, neither agree/disagree, disagree, strongly disagree

2. Most people I know usually vote in general elections.

Strongly agree, agree, neither agree/disagree, disagree, strongly disagree

3. I have a pretty good understanding of the important political issues facing our country.

Strongly agree, agree, neither agree/disagree, disagree, strongly disagree

4. It takes too much time and effort to be active in politics and public affairs.

Strongly agree, agree, neither agree/disagree, disagree, strongly disagree

5. Politicians don’t care what people like me think.

Strongly agree, agree, neither agree/disagree, disagree, strongly disagree

BESOP Big Five personality items (the Big Five items were not used to derive the satisficing indicators)

*The following are a number of statements that may or may not apply to you. Please rate each as whether it is very accurate, accurate, moderately accurate, neither inaccurate nor accurate, moderately inaccurate, inaccurate, or very inaccurate.*

I see myself as extroverted and enthusiastic (Extraversion).

I see myself as critical and quarrelsome (Agreeableness).

I see myself as dependable and self-disciplined (Conscientiousness).

I see myself as anxious and easily upset (Neuroticism).

I see myself as open to new experiences and complex (Openness).

I see myself as reserved and quiet (Extraversion).

I see myself as sympathetic and warm (Agreeableness).

I see myself as disorganized and careless (Conscientiousness).

I see myself as calm and emotionally stable (Neuroticism).

I see myself as conventional and uncreative (Openness).
