# Supplementary material for: Personality and Survey Satisficing
Source: Public Opin Q. 2023 Sep 17;87(3):689–718. doi: 10.1093/poq/nfad036 (PMC10662660; doi:10.1093/poq/nfad036)
Supplement: nfad036_Supplementary_Data [file nfad036_supplementary_data.pdf]

Personality and Survey Satisficing

Supplementary Material

Patrick Sturgis

London School of Economics

Ian Brunton-Smith

University of Surrey

Table S1: Logistic regression models predicting response style indicators – UKHLS CASI items only

	Straightlining						Don't knows						Midpoints					
	B	SE	Sig	B	SE	Sig	B	SE	Sig	B	SE	Sig	B	SE	Sig	B	SE	Sig
Agreeableness	-0.073	0.020	0.000	-0.089	0.020	0.000							-0.172	0.022	0.000	-0.179	0.023	0.000
Conscientiousness	-0.028	0.019	0.133	-0.038	0.019	0.043							-0.211	0.021	0.000	-0.190	0.021	0.000
Cognitive ability	-0.246	0.038	0.000	-0.144	0.042	0.001							-0.577	0.041	0.000	-0.498	0.049	0.000
Sex (female)				0.113	0.043	0.008										0.144	0.050	0.004
Interest in politics (ref= very)																		
Fairly interested				0.245	0.080	0.002										0.056	0.102	0.581
Not very interested				0.389	0.081	0.000										0.166	0.106	0.116
Not at all interested				0.482	0.084	0.000										0.338	0.107	0.002
Highest qualification (ref=degree)																		
Other degree				0.068	0.075	0.366										0.403	0.092	0.000
A-level				0.078	0.065	0.228										0.336	0.078	0.000
General Certificate of Secondary Education				0.181	0.064	0.005										0.463	0.080	0.000
Other qualification				0.151	0.084	0.071										0.601	0.100	0.000
No qualification				-0.032	0.091	0.728										0.437	0.108	0.000
Age (years)				0.006	0.001	0.000										-0.009	0.001	0.000
Constant	-1.718	0.128	0.000	-2.046	0.149	0.000							-0.562	0.140	0.000	-1.251	0.170	0.000
Unweighted N	34,065												34,065					

Table S2: Logistic regression models predicting response style indicators – UKHLS CAPI items only

	Straightlining						Don't knows						Midpoints					
	B	SE	Sig	B	SE	Sig	B	SE	Sig	B	SE	Sig	B	SE	Sig	B	SE	Sig
Agreeableness	-0.072	0.018	0.000	-0.057	0.019	0.002	-0.007	0.028	0.804	-0.018	0.028	0.510	-0.138	0.018	0.000	-0.136	0.019	0.000
Conscientiousness	0.007	0.018	0.700	0.015	0.018	0.418	-0.107	0.025	0.000	-0.034	0.026	0.182	-0.054	0.018	0.003	-0.017	0.019	0.368
Cognitive ability	-0.240	0.034	0.000	-0.199	0.040	0.000	-0.476	0.047	0.000	-0.373	0.060	0.000	-0.021	0.034	0.526	-0.036	0.040	0.370
Sex (female)				-0.273	0.038	0.000				0.414	0.059	0.000				-0.014	0.041	0.731
Interest in politics (ref= very)																		
Fairly interested				0.507	0.082	0.000				0.568	0.159	0.000				0.784	0.107	0.000
Not very interested				0.401	0.088	0.000				0.981	0.161	0.000				1.057	0.111	0.000
Not at all interested				0.342	0.091	0.000				1.698	0.158	0.000				1.078	0.110	0.000
Highest qualification (ref=degree)																		
Other degree				0.151	0.075	0.045				0.252	0.115	0.029				0.311	0.076	0.000
A-level				0.135	0.062	0.031				0.171	0.100	0.087				0.320	0.064	0.000
General Certificate of Secondary Education				0.233	0.065	0.000				0.406	0.098	0.000				0.383	0.067	0.000
Other qualification				0.224	0.081	0.006				0.577	0.119	0.000				0.346	0.088	0.000
No qualification				0.284	0.082	0.001				0.720	0.121	0.000				0.190	0.090	0.035
Age (years)				-0.001	0.001	0.321				-0.027	0.002	0.000				-0.014	0.001	0.000
Constant	-1.813	0.124	0.000	-2.354	0.145	0.000	-2.190	0.163	0.000	-4.289	0.225	0.000	-1.082	0.122	0.000	-2.475	0.166	0.000
Unweighted N	36,817						36,817						36,817					

Table S3: Logistic regression models predicting response style indicators (top 5%) – UKHLS

	Straightlining						Don't knows						Midpoints					
	B	SE	Sig	B	SE	Sig	B	SE	Sig	B	SE	Sig	B	SE	Sig	B	SE	Sig
Agreeableness	-0.114	0.024	0.000	-0.126	0.024	0.000	0.013	0.034	0.691	0.015	0.034	0.660	-0.209	0.030	0.000	-0.203	0.030	0.000
Conscientiousness	-0.062	0.024	0.011	-0.067	0.024	0.006	-0.167	0.033	0.000	-0.042	0.034	0.217	-0.242	0.027	0.000	-0.210	0.028	0.000
Cognitive ability	-0.286	0.049	0.000	-0.200	0.057	0.001	-0.533	0.058	0.000	-0.410	0.080	0.000	-0.375	0.054	0.000	-0.375	0.069	0.000
Sex (female)				0.060	0.057	0.287				0.521	0.081	0.000				0.061	0.063	0.331
Interest in politics (ref= very)																		
Fairly interested				0.380	0.112	0.001				0.084	0.283	0.765				0.442	0.166	0.008
Not very interested				0.414	0.113	0.000				0.938	0.281	0.001				0.634	0.175	0.000
Not at all interested				0.466	0.116	0.000				2.098	0.273	0.000				0.835	0.171	0.000
Highest qualification (ref=degree)																		
Other degree				0.183	0.100	0.067				0.386	0.172	0.025				0.423	0.129	0.001
A-level				0.108	0.088	0.218				0.125	0.148	0.397				0.203	0.113	0.074
General Certificate of Secondary Education				0.273	0.086	0.001				0.494	0.144	0.001				0.426	0.111	0.000
Other qualification				0.242	0.111	0.030				0.792	0.175	0.000				0.510	0.138	0.000
No qualification				0.059	0.121	0.623				0.845	0.172	0.000				0.224	0.152	0.142
Age (years)				0.004	0.002	0.035				-0.041	0.003	0.000				-0.014	0.002	0.000
Constant	-2.021	0.159	0.000	-2.485	0.189	0.000	-2.637	0.205	0.000	-5.578	0.344	0.000	-0.902	0.179	0.000	-2.050	0.243	0.000
Unweighted N	36,817						36,817						36,817					

Table S4: Logistic regression models predicting response style indicators (top 15%) – UKHLS

	Straightlining						Don't knows						Midpoints					
	B	SE	Sig	B	SE	Sig	B	SE	Sig	B	SE	Sig	B	SE	Sig	B	SE	Sig
Agreeableness	-0.058	0.016	0.000	-0.074	0.016	0.000	-0.015	0.027	0.585	-0.028	0.027	0.300	-0.195	0.017	0.000	-0.201	0.017	0.000
Conscientiousness	-0.015	0.014	0.300	-0.026	0.015	0.069	-0.104	0.024	0.000	-0.040	0.025	0.106	-0.174	0.016	0.000	-0.144	0.017	0.000
Cognitive ability	-0.153	0.029	0.000	-0.028	0.034	0.413	-0.511	0.046	0.000	-0.397	0.058	0.000	-0.242	0.031	0.000	-0.236	0.038	0.000
Sex (female)				0.059	0.033	0.079				0.405	0.057	0.000				0.136	0.038	0.000
Interest in politics (ref= very)																		
Fairly interested				0.345	0.065	0.000				0.512	0.149	0.001				0.448	0.086	0.000
Not very interested				0.431	0.067	0.000				0.897	0.151	0.000				0.568	0.089	0.000
Not at all interested				0.393	0.070	0.000				1.590	0.148	0.000				0.671	0.090	0.000
Highest qualification (ref=degree)																		
Other degree				0.140	0.060	0.019				0.190	0.113	0.094				0.321	0.072	0.000
A-level				0.151	0.052	0.004				0.146	0.098	0.137				0.303	0.061	0.000
General Certificate of Secondary Education				0.301	0.050	0.000				0.365	0.097	0.000				0.486	0.062	0.000
Other qualification				0.257	0.065	0.000				0.540	0.116	0.000				0.523	0.080	0.000
No qualification				0.096	0.069	0.167				0.688	0.117	0.000				0.324	0.084	0.000
Age (years)				0.007	0.001	0.000				-0.024	0.002	0.000				-0.014	0.001	0.000
Constant	-1.339	0.100	0.000	-1.733	0.118	0.000	-2.118	0.158	0.000	-4.005	0.213	0.000	-0.049	0.110	0.652	-1.107	0.142	0.000
Unweighted N	36,817						36,817						36,817					

Table S5: Logistic regression models predicting response style indicators (top 5%) – BESOP

	Straightlining						Don't knows						Midpoints					
	B	SE	Sig	B	SE	Sig	B	SE	Sig	B	SE	Sig	B	SE	Sig	B	SE	Sig
Agreeableness	-0.085	0.023	0.000	-0.077	0.025	0.002	-0.007	0.027	0.790	-0.008	0.031	0.808	-0.061	0.024	0.010	-0.056	0.024	0.020
Conscientiousness	-0.141	0.021	0.000	-0.101	0.023	0.000	-0.076	0.026	0.004	-0.022	0.028	0.437	-0.082	0.021	0.000	-0.071	0.021	0.001
Sex (female)				0.328	0.096	0.001				0.712	0.124	0.000				-0.125	0.088	0.154
Attention to politics				-0.205	0.017	0.000				-0.410	0.021	0.000				-0.110	0.015	0.000
Highest qualification (ref=postgraduate degree)																		
Undergraduate degree				0.079	0.200	0.692				0.082	0.249	0.741				-0.070	0.170	0.680
A-level				0.292	0.201	0.146				0.376	0.239	0.116				-0.033	0.181	0.855
General Certificate of Secondary Education				0.622	0.202	0.002				0.490	0.243	0.044				0.267	0.182	0.144
Below General Certificate of Secondary Education				0.938	0.245	0.000				0.451	0.299	0.131				0.292	0.226	0.197
No qualification				0.687	0.236	0.004				0.581	0.289	0.044				-0.068	0.214	0.749
Age (years)				-0.038	0.003	0.000				-0.037	0.004	0.000				-0.005	0.003	0.062
Constant	-1.345	0.185	0.000	0.600	0.292	0.039	-2.725	0.192	0.000	-0.158	0.324	0.626	-2.071	0.201	0.000	-1.233	0.285	0.000
Unweighted N	24980						25073						25073					

Table S6: Logistic regression models predicting response style indicators (top 15%) – BESOP

	Straightlining						Don't knows						Midpoints					
	B	SE	Sig	B	SE	Sig	B	SE	Sig	B	SE	Sig	B	SE	Sig	B	SE	Sig
Agreeableness	-0.017	0.014	0.211	-0.017	0.015	0.268	0.041	0.015	0.005	0.016	0.017	0.356	-0.032	0.015	0.027	-0.028	0.015	0.060
Conscientiousness	-0.085	0.013	0.000	-0.046	0.014	0.001	-0.076	0.014	0.000	-0.040	0.016	0.011	-0.066	0.014	0.000	-0.057	0.015	0.000
Sex (female)				0.354	0.058	0.000				0.815	0.064	0.000				-0.087	0.058	0.134
Attention to politics				-0.242	0.011	0.000				-0.346	0.012	0.000				-0.113	0.010	0.000
Highest qualification (ref=postgraduate degree)																		
Undergraduate degree				0.288	0.116	0.013				0.313	0.125	0.012				-0.175	0.103	0.089
A-level				0.375	0.120	0.002				0.429	0.128	0.001				-0.127	0.110	0.249
General Certificate of Secondary Education				0.819	0.120	0.000				0.546	0.128	0.000				0.032	0.109	0.770
Below General Certificate of Secondary Education				1.071	0.157	0.000				0.632	0.159	0.000				0.056	0.145	0.701
No qualification				0.986	0.140	0.000				0.674	0.146	0.000				-0.167	0.132	0.206
Age (years)				-0.033	0.002	0.000				-0.021	0.002	0.000				-0.004	0.002	0.053
Constant	-0.876	0.114	0.000	1.058	0.182	0.000	-1.601	0.111	0.000	0.353	0.188	0.060	-1.361	0.122	0.000	-0.430	0.172	0.012
Unweighted N	24980						25073						25073					

Table S7: Causal sensitivity analysis results – UKHLS[1]

	Agreeableness			Conscientiousness		
	B	SE	Sig	B	SE	Sig
Straightlining						
Original estimate	-0.009	0.002	0.000	-0.003	0.002	0.026
1x cognitive ability	-0.008	0.002	0.000	-0.003	0.002	0.037
2x cognitive ability	-0.008	0.002	0.000	-0.003	0.002	0.053
3x cognitive ability	-0.008	0.002	0.000	-0.003	0.002	0.074
Midpoints						
Original estimate	-0.015	0.001	0.000	-0.013	0.001	0.000
1x cognitive ability	-0.015	0.001	0.000	-0.013	0.001	0.000
2x cognitive ability	-0.014	0.001	0.000	-0.012	0.001	0.000
3x cognitive ability	-0.014	0.001	0.000	-0.011	0.001	0.000
Unweighted N		36,817			36,817	

Notes: [1] Estimated with the R sensemakr package using a linear probability model.

Table S8: Causal sensitivity analysis results – BESOP[1]

	Agreeableness			Conscientiousness		
	B	SE	Sig	B	SE	Sig
Straightlining						
Original estimate	-0.004	0.001	0.001	-0.006	0.001	0.000
1x political attentiveness	-0.004	0.001	0.001	-0.005	0.001	0.000
2x political attentiveness	-0.004	0.001	0.001	-0.004	0.001	0.000
3x political attentiveness	-0.004	0.001	0.001	-0.003	0.001	0.002
Don't knows						
Original estimate				-0.003	0.001	0.001
1x political attentiveness				-0.002	0.001	0.033
2x political attentiveness				-0.001	0.001	0.341
3x political attentiveness				0.0003	0.001	0.721
Midpoints						
Original estimate	-0.003	0.001	0.004	-0.005	0.001	0.000
1x political attentiveness	-0.003	0.001	0.005	-0.005	0.001	0.000
2x political attentiveness	-0.003	0.001	0.005	-0.005	0.001	0.000
3x political attentiveness	-0.003	0.001	0.005	-0.004	0.001	0.000
Unweighted N		25073			25073	

Notes: [1] Estimated with the R sensemakr package using a linear probability model.
